# Drug resistance and pathogenicity characteristics of *Escherichia coli* causing pneumonia in farmed foxes

**DOI:** 10.3389/fvets.2025.1567009

**Published:** 2025-04-09

**Authors:** Chunxiao Zhang, Hong Li, Qi Zhao, Lili Wang, Guanxin Hou, Qiumei Shi, Tonglei Wu, Guangping Gao, Zhiqiang Zhang

**Affiliations:** ^1^Hebei Key Laboratory of Preventive Veterinary Medicine, Hebei Normal University of Science and Technology, Qinhuangdao, China; ^2^College of Veterinary Medicine, Hebei Agricultural University, Baoding, China

**Keywords:** fox, pneumonia, *Escherichia coli*, multi-drug resistance, multilocus sequence typing

## Abstract

Bacterial pneumonia is a leading cause of mortality in fur-bearing animals, posing significant threat to fur production. To clarify the pathogenic agent of bacterial pneumonia in farmed foxes from eastern Hebei province, China, we performed bacterial isolation and identification from samples between 2020 and 2023. A total of 142 bacterial strains were isolated, of which 101 were identified as *Escherichia coli* (*E. coli*), indicating that *E. coli* is the major cause responsible for bacterial pneumonia in farmed foxes. Serotyping identification showed that a total of 8 serotypes were prevalent in the *E. coli* isolates, with O1, O8, O78 and O12 being the dominant ones. Five *E. coli* isolates were randomly picked for pathogenicity testing, and all of them were able to cause pneumonia symptoms in 6-week-old Kunming mice, accompanied by organ damage in lung. Eleven virulence genes were demonstrated present among the *E. coli* isolates. Antibiotic susceptibility tests showed that 78 of 101 *E. coli* strains exhibited multi-drug resistance (MDR), with the highest resistance rates against tetracyclines, and some strains showed resistance to carbapenems. Notably, no single antibiotic was effective against all strains. Twenty antibiotic resistance genes (ARGs) were detected among the isolates. Multilocus sequence typing (MLST) revealed 11 sequence types (STs) among 19 *E. coli* isolates, with ST-101 predominating (4/19). These findings enhance our understanding of the epidemiology, resistance traits, and pathogenicity of fox-derived pathogenic *E. coli* in Hebei.

## 1 Introduction

Bacterial pneumonia is a prevalent and recurrent disease in fur-bearing animal farming, serving as the leading cause of mortality during the growth phase (1–3). This disease primarily affects young animals aged 4 to 6 months and is particularly endemic from August to October, resulting in significant mortality rates (4, 5). In minks, *Pseudomonas aeruginosa* (*P. aeruginosa*) has been reported as the primary pathogen responsible for hemorrhagic pneumonia (1, 4, 6, 7); as for other fur-bearing animals, the etiology is complex, with involvement of multiple pathogens, such as *Escherichia coli* (*E. coli*), *Klebsiella pneumoniae* (*KP*), and *P. aeruginosa* (7, 8).

*E. coli* can cause a variety of infections in humans and animals, mainly manifesting as gastrointestinal diseases (9–13). While in farmed foxes, raccoon dogs, and minks, *E. coli* is associated with diverse severe health issues, including acute hemorrhagic pneumonia and systemic infections (3, 7, 8). Currently, there are commercial vaccines available for *P. aeruginosa*-induced pneumonia in minks, but due to the complex serotype diversity of *E. coli*, no commercial vaccine is available (14). Consequently, antibiotics remain the primary method for controlling pulmonary infections; however, the increasing prevalence of antibiotic resistance among bacterial strains presents significant challenges for disease management (15–17).

The eastern region of Hebei province is one of the three main breeding areas for fur animals in China, with fox and raccoon being the major breeding species. And the fur-bearing animals here, especially foxes, have been suffering from the severe threat of bacterial pneumonia in recent years. To elucidate the pathogenic characteristics of bacterial pneumonia in foxes within Hebei Province, this study focused on isolating and identifying pathogens from foxes with pneumonia between 2020 and 2023, and further investigating the antibiotic resistance and pathogenicity of the isolates to provide clinical guidance for the prevention and control of this disease.

## 2 Materials and methods

### 2.1 Sample collection and identification of bacteria

In this study, we collected 142 samples of fox pneumonia cases from 98 farms in eastern Hebei Province, China, between 2020 and 2023. The collected samples, including lungs and blood were transported to the laboratory within 24 h in ice packs for bacterial isolation and culture. First, the samples were inoculated onto a 5% defibrinated sheep blood agar medium (Qingdao Haibo Biotechnology Co., Ltd., Qingdao, China), followed by incubation at 37°C for 12 h in a constant temperature incubator. After purification, the isolates were subjected to Gram staining (Beijing Solarbio Science & Technology Co., Ltd., Beijing, China) and 16S rDNA sequence analysis.

### 2.2 Serotype identification

To expose the O antigen for detection, the bacterial suspension was autoclaved for 2 h to remove the K antigen on the bacterial surface. Then, sera specific to various *E. coli* O antigens (Beijing Zhonghai Biotech Co., Ltd., Beijing, China) were performed slide agglutination tests for the serotyping of isolated bacteria. A clear agglutination reaction (marked as ++) within 30 s was considered the standard for a positive result. The procedure was performed according to the manufacturer’s instructions.

### 2.3 Pathogenicity testing of *Escherichia coli* isolates in mice

Five *E. coli* isolates of different serotypes (The serotypes of the five strains were O1, O8, O78, O12, and O9, respectively) were tested for pathogenicity in 6-week-old Kunming mice, purchased from SPF (Beijing, China) Biotechnology Co., Ltd. Pathogenicity testing followed Zhang et al. (8). Sixty mice were randomly divided into 6 groups with 10 mice per group. The isolates were cultured to logarithmic growth phase and then diluted to a concentration of 1 × 10^7^ CFU/mL with phosphate buffered saline (PBS). The Kunming mice were intraperitoneally challenged with 0.2 mL bacterial suspension or PBS as control, and the infected mice were continuously observed for 7 d. The clinical symptoms, pathologic variation, and mortality were recorded, and *E. coli* was reisolated and identified from the lungs of the infected mice. During the experiment, mice were euthanized to observe the pathological changes at 5-days postinfection (dpi), and one healthy mouse was randomly selected from the control group, followed by pathologic alterations being observed and compared. And the lung tissues of mice were taken and fixed in 3.5% neutral buffered formalin solution and further made into pathological sections for histopathological analysis.

### 2.4 Detection of virulence genes

DNA was extracted from the isolated strains by bacterial genome extraction kit (Beijing Solarbio Science & Technology Co., Ltd., Beijing, China). In total, 11 virulence genes previously reported were further identified by PCR, including *vat, iutA*, *Iss, hlyF*, *iucD*, *tsh*, *cvaA/B*, *cvaC, trat, ECs3737,* and *ECs3703.* The primer sequences and amplified fragment sizes are shown in Supplementary Table 1, and the primers were synthesized by Sangon Bioengineering (Shanghai, China) Co., Ltd.

### 2.5 Antibiotic sensitivity test

The antibiotic sensitivity of the *E. coli* isolates to 25 antibiotics was assayed by the Kirby-Bauer disk diffusion method according to the Clinical and Laboratory Standards Institute (CLSI) standards. The bacterial culture was diluted to 0.5 McFarland units with normal saline and spread on the Mueller-Hinton agar medium. Then the antibiotic disks (Hangzhou Microbial Reagent Co., Ltd., Hangzhou, China) were placed on the surface of the Mueller-Hinton agar medium and cultured in a constant temperature incubator at 37°C for 16–18 h. The diameter of the inhibition zone of each strain was measured, and based on the CLSI standards, the drug susceptibility test results were judged in three forms: sensitivity (S), intermediate (I), and resistance (R). *E. coli* ATCC 25922 was used as the quality control strain. Multi-drug resistance (MDR) strains were defined as those resistance to three or more antimicrobial classes (18).

### 2.6 Detection of antibiotic resistance genes

In this study, carriage of 25 antibiotic resistance genes (ARGs) in the *E. coli* isolates was further determined by PCR, including aminoglycoside resistance genes (*strA*, *aadA2*, *aac2*, *aac4*), fluoroquinolones resistance genes (*gyrA*, *QnrS*, *qnrD*, *qnrC*, *qepA*, *qnrA*, *qnrB*, *gyrB*), *β*-lactam resistance genes (*CMY-2*, *CTX-M*, *SHV*), amphenicol resistance gene (*floR*), macrolide resistance gene (*mph(A)*), tetracycline resistance genes (*tet(A)*, *tet(B)*, *tet(E)*), fosfomycin resistance gene (*fosA*), sulfonamides resistance genes (*sul*, *sul2*), and the Class 1 and Class 2 integrons (*int1*, *int2*). The primer sequences and amplified fragment sizes are shown in Supplementary Table 2, and some primers were designed according the literature previously reported (14) and synthesized by Sangon Bioengineering (Shanghai, China) Co., Ltd.

### 2.7 Multilocus sequence typing

To investigate the genetic diversity and epidemiological links of the isolates, 19 *E. coli* strains were randomly selected for multilocus sequence typing (MLST). The primer sequences for the seven housekeeping genes of *E. coli* (*adk*, *fumC*, *icd*, *purA*, *gyrB*, *recA*, and *mdh*) were obtained from http://enterobase.warwick.ac.uk/species/ecoli/allele_st_search (14, 19). The primer sequences, fragment sizes, and annealing temperatures are shown in Supplementary Table 3. And the sequencing work of the experiment was completed by Shanghai Hongxu Biotechnology (Shanghai, China) Co., Ltd. After sequencing was completed, cluster analysis was performed using PHYLOViZ software, and a Venn diagram was constructed following the methodology described in Peng et al. (20) to illustrate the shared and unique sequence types (STs) between human commensal *E. coli* and fox-derived *E. coli*.

## 3 Results

### 3.1 Strain source and identification

**Figure 1:**
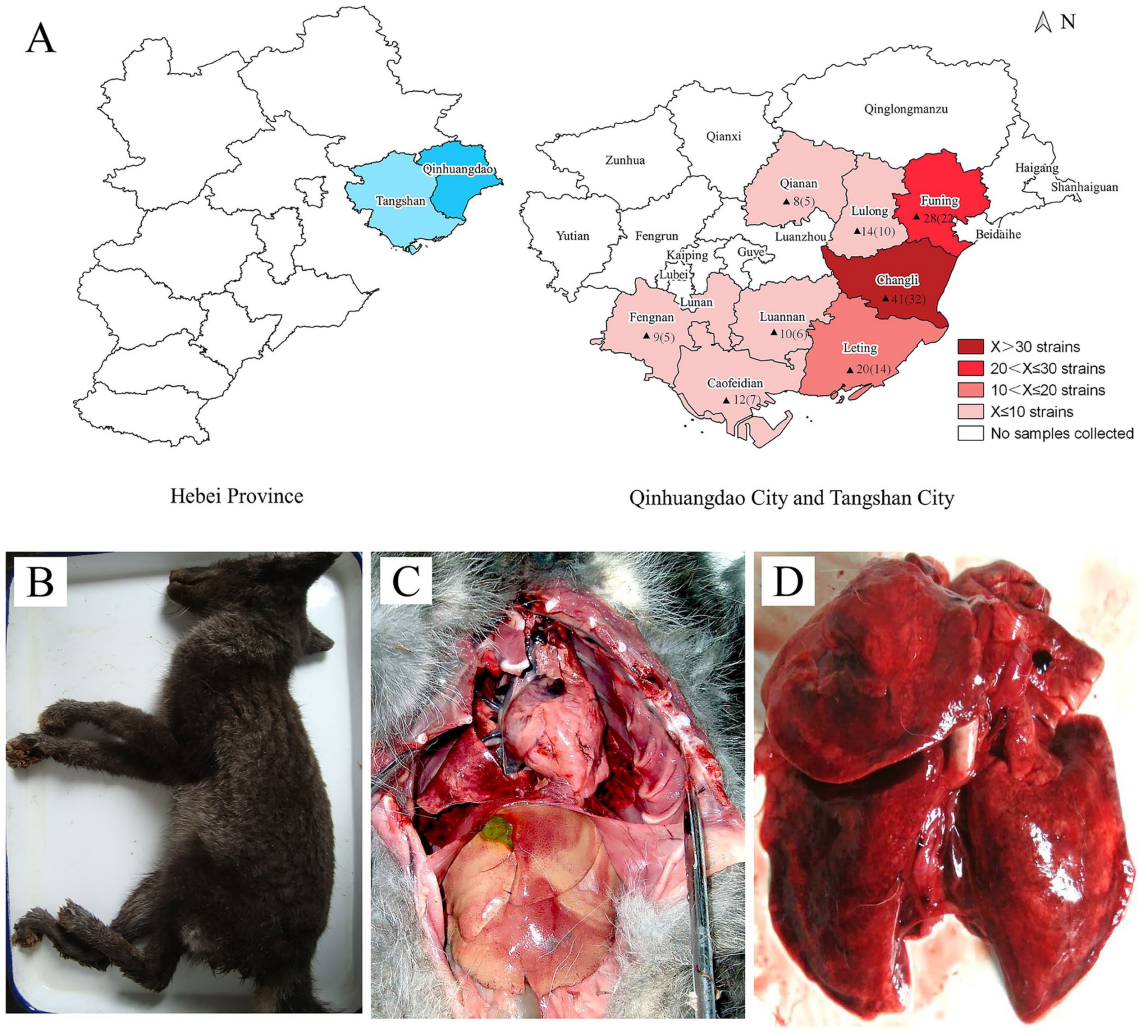
Geographical distribution of *E. coli* isolates and pathological observations of *E. coli*-infected fox. **(A)** Geographical distribution of samples and *E. coli* isolates. The sample-sourced region is marked in red, and the number of samples and *E. coli* isolates is labeled in the corresponding region, with the latter indicated in parentheses. **(B)** *E. coli*-infected fox. **(C)** Multi-organ pathological observations of fox. **(D)** Pathological observation of lungs in fox.

From 2020 to 2023, respiratory diseases occurred frequently in foxes at fur-bearing animal farms in the eastern region of Hebei Province, leading to mortality and economic losses. To identify the pathogens causing pneumonia, samples were collected from the affected foxes, and isolation and identification of the pathogens were conducted. A total of 142 dominant bacterial strains were isolated, and further identified by 16S rDNA sequencing. Of these strains, 101 were identified as *E. coli*, 15 as *Pasteurella*, 13 as *KP*, 8 as *P. aeruginosa*, 3 as *Streptococcus*, and 2 as *Staphylococcus*, suggesting that *E. coli* is the major, but not the only, etiologic agent responsible for bacterial pneumonia in farmed foxes. Figure 1A illustrates the geographical distribution of samples and *E. coli* isolates. Clinically, symptoms such as depression, lethargy, dyspnea, and diarrhea are exhibited by foxes infected with *E. coli*. Necropsy findings often include acute hemorrhagic pneumonia and systemic infection, with particularly evident lung lesions (Figures 1B–D).

### 3.2 Serotype identification

To identify the serotypes of pathogenic *E. coli*, slide agglutination tests were performed on 101 isolated strains. The data showed that, a total of 98 isolates were successfully serotyped with 3 isolates untyped, 8 distinct serotypes were determined present in the *E. coli* isolates, of which O1 (26/101), O8 (19/101), O78 (17/101), and O12 (17/101), were predominant serotypes (Table 1).

**Table 1:** Detection of serotypes.

Serotype	Number of positives/Total number of samples	Detection rate (%)
O_1_	26/101	25.7
O_8_	19/101	18.8
O_78_	17/101	16.8
O_12_	17/101	16.8
O_9_	9/101	8.9
O_2_	7/101	6.9
O_6_	5/101	5.0
O_15_	1/101	1.0

### 3.3 The *Escherichia coli* isolates showed high pathogenicity to mice

To assess the pathogenicity of *E. coli* isolates, five strains were randomly selected for pathogenicity assessment. The data showed that, the mice received *E. coli* infection at a dose of 2 × 10^6^ CFU exhibited apparent clinical symptoms such as ruffled fur, hunched posture, and anorexia. The mice began to suffer deaths at 3 dpi, with a cumulative mortality rate of 100% by day 7. The pathogenicity of the five strains showed no significant differences. In contrast, no abnormalities were observed in the control group. The isolate-like strains were re-isolated from the deceased mice.

**Figure 2:**
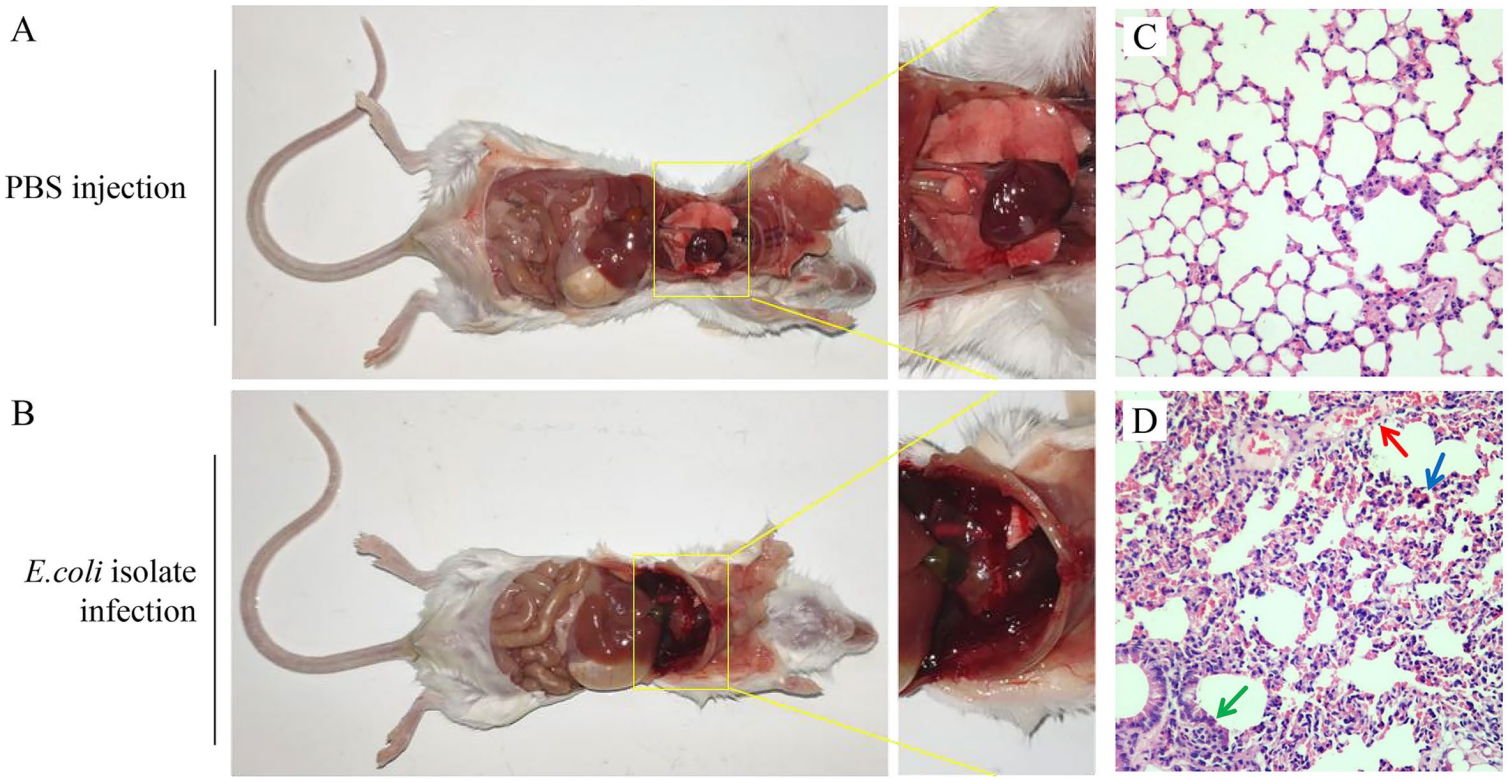
Pathological observations and histopathological analysis of infected mice. **(A)** Mouse in the control group. **(B)** Mouse in the experimental group. **(C)** Lung sample was collected from healthy mouse for pathological sectioning and hematoxylin–eosin staining. The histopathological changes were observed under a microscope (200×). **(D)** Lung sample was collected from *E. coli*-infected mouse for pathological sectioning and hematoxylin–eosin staining. The histopathological changes were observed under a microscope (200×).

These findings suggest that the 5 *E. coli* isolates are highly pathogenic to mice. Pathological observations revealed congestion and hemorrhage in the lungs of deceased mice, indicating significant pathological changes (Figures 2A,B).

Further histopathological analysis showed that, compared with the control group, the experimental group of mice revealed significant thickening of alveolar walls (green arrow) and diffuse erythrocyte infiltration in interstitial tissues (red arrow), with extensive inflammatory cell infiltration (blue arrow) (Figures 2C,D). Figure 2 shows the pathological results of mouse infection caused by the strain with serotype O1.

### 3.4 The *Escherichia coli* isolates carried multiple virulence genes

Considering the high pathogenicity of *E. coli* isolates, we detected the prevalence of 11 virulence genes using PCR. All targeted genes were found among the isolates, with the highest carrying rate of *ECs3737* and *ECs3703* genes at 63.4 and 58.4%, respectively, followed by the virulence genes *trat*, *iutA*, and *iucD*, with rates of 38.6, 28.7, and 27.7%, respectively (Table 2).

**Table 2:** Detection of virulence genes.

Virulence gene	Number of positives/Total number of samples	Detection rate (%)
Vat	6/101	5.9
iutA	29/101	28.7
Iss	14/101	13.9
hlyF	22/101	21.8
iucD	28/101	27.7
tsh	6/101	5.9
cvaA/B	13/101	12.9
cvaC	11/101	10.9
trat	39/101	38.6
ECs3737	64/101	63.4
ECs3703	59/101	58.4

### 3.5 The *Escherichia coli* isolates displayed severe resistances to multiple antibiotics

**Figure 3:**
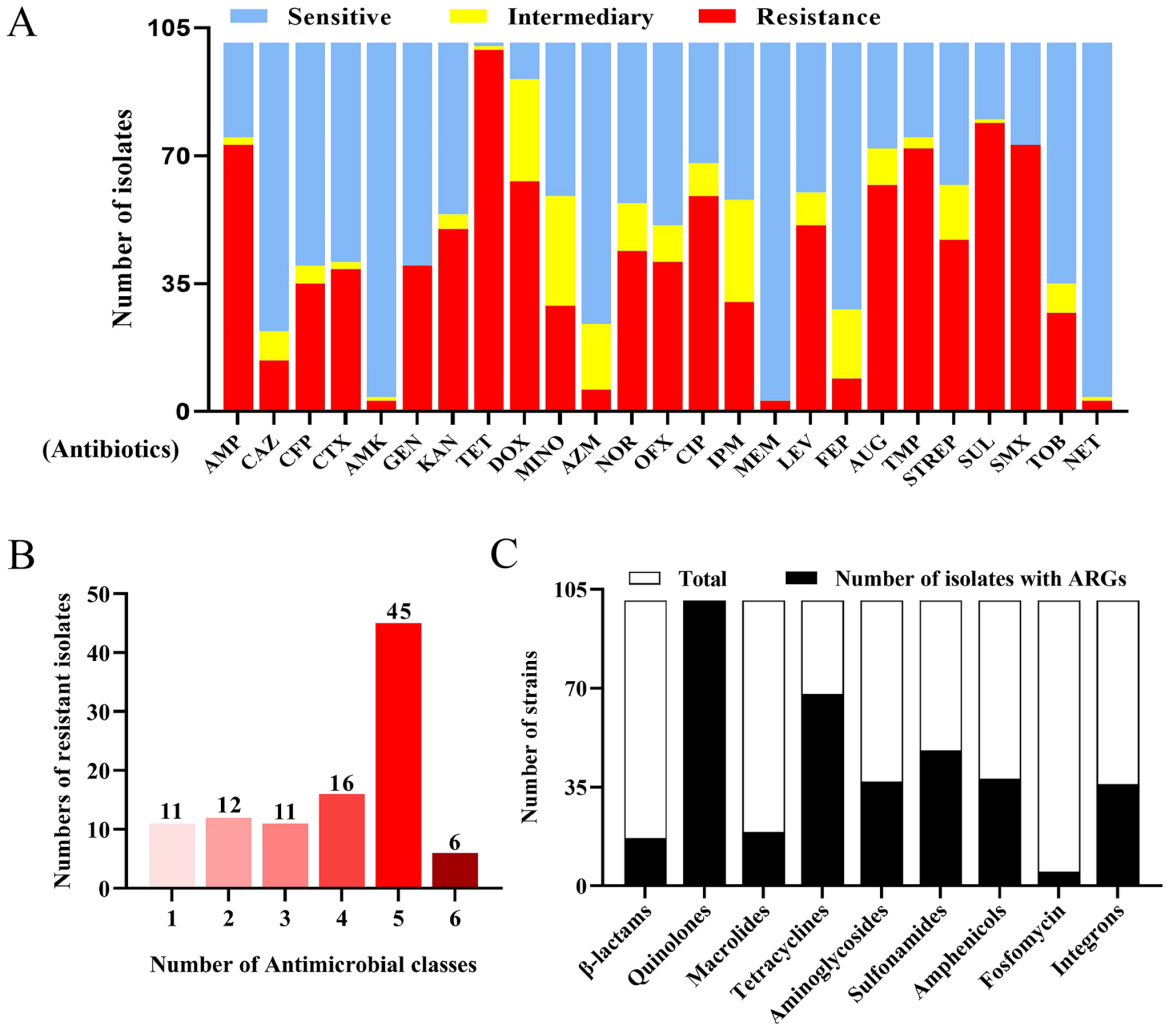
Drug resistance characteristics of 101 *E. coli* isolates. **(A)** Sensitivity, moderate sensitivity, and resistance ratios of *E. coli* isolates to 25 antibiotics. The ratios were calculated by dividing the number of isolates showing sensitivity, intermediate, and resistance to a certain antibiotic by the total number of 101. ampicillin (AMP); Ceftazidime (CAZ); Cefoperazone (CFP); Cefotaxime (CTX); Amikacin (AMK); Gentamicin (GEN); Kanamycin (KAN); Tetracycline (TET); Doxycycline (DOX); Minocycline (MINO); Azithromycin (AZM); Norfloxacin (NOR); Ofloxacin (OFX); Ciprofloxacin (CIP); Imipenem (IPM); Meropenem (MEM); Levofloxacin (LEV); Cefepime (FEP); Augmentin (AUG); Trimethoprim (TMP); Streptomycin (STREP); Sulfisoxazole (SUL); Sulfamethoxazole (SMX); Tobramycin (TOB); Netilmicin (NET). **(B)** Numbers of *E. coli* isolates resistant to different antibiotic classes tested. The antibiotic classes include *β*-Lactams, Aminoglycosides, Tetracyclines, Macrolides, Quinolones, and Sulfonamides. **(C)** Detection of ARGs in *E. coli.*

To elucidate the antibiotic resistance patterns of fox-derived *E. coli*, antibiotic susceptibility testing was conducted on 101 *E. coli* isolates against 25 antibiotics. The results revealed a highly complex antibiotic resistance profile with significant variation among strains; 13 isolates showed the most severe drug resistance by resistance to 16 antibiotics, accounting for 12.9% (13/101). An additional table shows this in more detail (see Supplementary Table 4). The highest resistance rate was observed for tetracycline (98.0%), followed by sulfisoxazole (78.3%), ampicillin (72.3%), and sulfamethoxazole (72.3%). Notably, no single antibiotic was effective against all strains (Figure 3A). Statistical analysis revealed that 78 strains were resistant to three or more classes of antibiotics, exhibiting varying degrees of MDR, with an MDR rate of 77.3% (Figure 3B).

### 3.6 The *Escherichia coli* isolates carried multiple ARGs

The carriage and horizontal transfer of ARGs are the main reasons for bacteria developing antibiotic resistance. Here, 25 ARGs were determined by PCR, and a total of 20 ARGs were detected in 101 *E. coli* isolates (Table 3). It is noteworthy that all types of resistance genes were detected among the 101 *E. coli* strains. Specifically, 16.8% (17/101) harbored *β*-lactam resistance genes, while 100% (101/101) possessed fluoroquinolone resistance genes. Macrolide resistance genes were identified in 18.8% (19/101), tetracycline resistance genes in 67.3% (68/101), aminoglycoside resistance genes in 36.6% (37/101), amphenicol resistance genes in 37.6% (38/101), sulfonamide resistance genes in 47.5% (48/101), fosfomycin resistance genes in 5% (5/101), and integron genes in 35.6% (36/101) of the strains (Figure 3C).

**Table 3:** Detection of resistance genes.

antibiotic classes	Gene	Number of positives/Total number of samples	Detection rate (%)
*β*-Lactams	CMY-2	2/101	2.0
	CTX-M	17/101	16.8
	SHV	0/101	0
Quinolones	gyrA	101/101	100
	QnrS	12/101	11.9
	qnrD	0/101	0
	qnrC	0/101	0
	qepA	1/101	1.0
	qnrA	0/101	0
	qnrB	5/101	5.0
	gyrB	93/101	92.1
Macrolides	mph(A)	19/101	18.8
Tetracyclines	tetB	31/101	30.7
	tetA	68/101	67.3
	tetE	0/101	0
Aminoglycosides	strA	37/101	36.6
	aadA2	33/101	32.7
	aac2	8/101	7.9
	aac4	8/101	7.9
Sulfonamides	sul	41/101	40.6
	sul2	48/101	47.5
Amphenicols	floR	38/101	37.6
fosfomycin	fosA	5/101	5.0
integrons	int1	36/101	35.6
	Int2	2/101	2.0

### 3.7 The fox-derived *Escherichia coli* isolates displayed partial same MLSTs with that of human originated isolates

**Figure 4:**
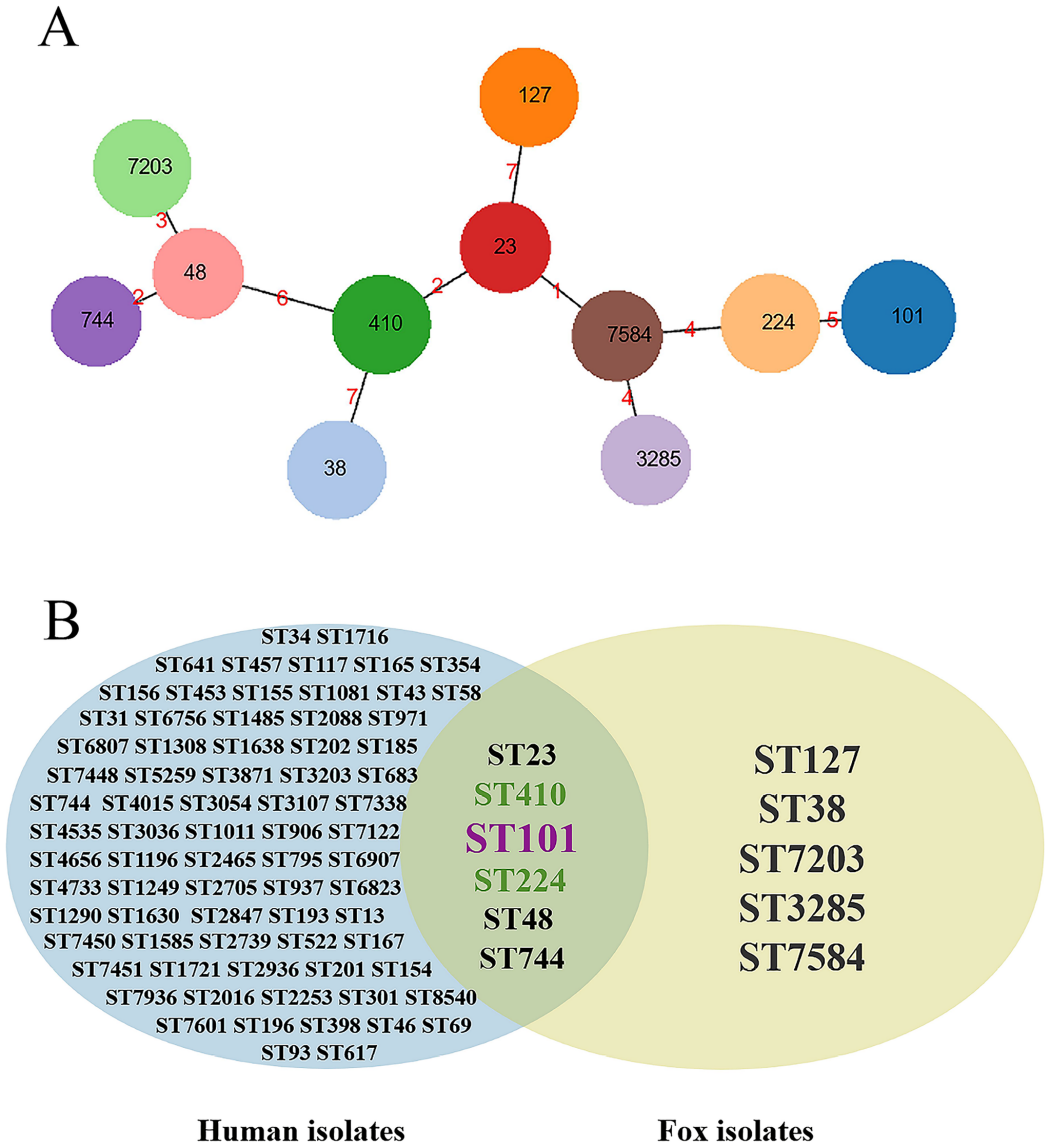
Cluster analysis and Venn diagram. **(A)** Cluster analysis was performed by PHYLOViZ software v1.1.5. The black number represents ST types; the red number represents the difference in allele numbers between different ST types. **(B)** Venn diagram showing shared and unique STs between human commensal *E. coli* and fox-derived *E. coli*.

Following MLST analysis of 19 *E. coli* strains and subsequent cluster analysis using PHYLOViZ software, 11 distinct STs were identified. ST101 was the most prevalent, representing 21.1% of the strains. ST410, ST127, ST38, ST224, and ST7203 each accounted for 10.5%, while ST744, ST3285, ST7584, ST23, and ST48 were less common, each detected in 5.3% of the strains (Figure 4A). Details of *E. coli* strains that underwent MLST are presented in Supplementary Table 5. To determine the genetic propensity of drug-resistant isolates to spread into the human sector, a Venn diagram was constructed to compare shared and unique STs between human commensal *E. coli* and fox-derived *E. coli*. The analysis identified 6 STs that were common to both sources: ST101, ST410, ST224, ST23, ST48, and ST744 (Figure 4B).

## 4 Discussion

Hemorrhagic pneumonia is one of the leading causes of mortality in fur-bearing animals, commonly occurring in the autumn months and presenting with rapid onset and swift mortality, making it a significant challenge for fur-bearing animal production (21). Although *P. aeruginosa* has been determined as the primary pathogen responsible for hemorrhagic pneumonia (22), recent studies have reported the close relationship between *E. coli* and pneumonia outbreaks in farmed fur-bearing animals (3).

As a major province for fur-bearing animal breeding, the farming area is mainly focused at Qinhuangdao and Tangshan City, with fox and raccoon dogs as the most important species. From 2020 to 2023, outbreaks of hemorrhagic pneumonia were frequently reported in farmed foxes in the eastern region of Hebei Province. In the present study, we performed bacterial isolation and identification from pneumonia-affected foxes, and recovered a total of 142 bacterial strains. Among these isolates, 101 were identified as *E. coli*, while the remaining 41 strains were identified as *Pasteurella*, *KP*, *P. aeruginosa*, *Streptococcus*, and *Staphylococcus*. It is noteworthy that *P. aeruginosa*, the primary pathogen responsible for hemorrhagic pneumonia in mink, has also been detected in foxes, demonstrating its capacity to cause hemorrhagic pneumonia in this species. However, the low detection rate observed in this study suggests that *P. aeruginosa* is not the predominant pathogen underlying bacterial pneumonia in foxes. Pathogenicity tests demonstrated that the isolates could replicate pneumonia-like symptoms in a mouse model, indicating that *E. coli* is a predominant pathogen responsible for pneumonia in foxes in this region. *E. coli* is known to cause various types of infections, and there are numerous reports regarding its ability to induce severe pneumonia in humans as well as in animals such as horses, dogs, and cats (23–26). However, the literature about *E. coli*-induced infections in fur-bearing animals is limited. Similar to infections caused by *P. aeruginosa*, *E. coli* infections in foxes frequently lead to hemorrhagic pneumonia. In this study, the data of pathogenicity testing revealed that the isolates displayed high pathogenicity to mice, with severe hemorrhage and damage in the lungs.

The virulence factors contribute to the pathogenicity of *E. coli* infections (27, 28), and we examined the virulence factors of clinical isolates of *E. coli* and found that *ECs3737* (63.4%), *ECs3703* (58.4%), and *trat* (38.6%) had the highest detection rates. ECs3703 and ECs3737 genes are the marker genes for the virulence island of *E. coli* type III secretion system 2 (ETT2), suggesting that ETT2 plays a key role in *E. coli* causing bacterial pneumonia in foxes (29–31). The *trat* gene from the tra manipulator could confer bacteria the ability to escape from serum-killing (32), which is critical to bacterial septic infection, and the high detection rate of this gene may be an important explanation for *E. coli*’s susceptibility to cause septic infections in fur-bearing animals.

The development of a vaccine against *E. coli* remains under experimental stage due to complex serotypes. Here we serotyped *E. coli* isolates from Hebei, and identified four dominant serotypes. These serotypes partially overlapped with those reported in other regions and animal species (33–35), while maintaining distinct regional features. This finding provides guidance for vaccine development. Antibiotics remain the primary strategy for dealing with bacterial pneumonia in fur-bearing animals (3), but the increasing bacterial antibiotic resistance posed a considerable challenge to both the treatment of this disease and to human health (36). We examined the antibiotic susceptibility of the 101 *E. coli* isolates, and found that the isolates exhibited severe MDR, with the most severe resistance to tetracycline with a 98% resistance rate, and a high resistance rate to ampicillin and sulfonamides, which was consistent with a previous report (7). Notably, none of the 25 antibiotics tested proved to be sensitive to all the isolates, indicating the severe resistance of pathogenic *E. coli* in the Hebei region.

The bacterial antibiotic resistance in livestock production has become an increasing concern (37–39), and the isolates carrying plasmids targeting third-generation cephalosporins and carbapenem resistance genes from farm animals have been reported recently (40). In this study, some isolates were determined to exhibit resistance to the carbapenem antibiotics imipenem and meropenem, with a resistance rate of 29.7 and 3%, respectively, whereas both antibiotics are not approved to be used in farmed animals. The emergence of carbapenem-resistant *E. coli* from farmed animals would pose a significant threat to human health (41). The carriage and transmission of ARGs are the basis for the bacterial resistance (42, 43), and we examined the carriage of ARGs in 101 *E. coli* isolates and found that all the ARGs, including *β*-lactams, aminoglycosides, quinolones, amphenicol, macrolides, tetracyclines, sulfonamides, and fosfomycin resistance genes, as well as integrons, were determined prevalent. Among which, the detection rate of quinolone resistance genes was 100%, followed by tetracyclines (67.3%) and sulfonamides resistance genes (47.5%), which was consistent with the results of resistance phenotype detection. Integrons are considered to be a significant cause for the transmission and development of bacterial drug resistance (44–46), and in this study, a 35.6% carrier rate of class 1 integrons was determined among the *E. coli* isolates.

MLST analysis is a bacterial typing method based on housekeeping genes (47), and here we randomly picked 19 fox-derived *E. coli* isolates for MLST analysis, and identified a total of 11 STs. Among these, 6 STs were also the types found in human commensal *E. coli*, suggesting that fox-derived *E. coli* may pose a threat to human health.

In sum, we isolated and identified the causative pathogens from foxes suffering from pneumonia in the eastern region of Hebei Province from 2020 to 2023, and demonstrated that *E. coli* is the major etiological agent responsible for hemorrhagic pneumonia in foxes in this region. These *E. coli* isolates exhibited high pathogenicity and severe MDR, posing a potential threat to fox farming and human health.

## Data Availability

The original contributions presented in the study are included in the article/Supplementary material, further inquiries can be directed to the corresponding author.
