# Supplementary material for: Drug resistance and pathogenicity characteristics of Escherichia coli causing pneumonia in farmed foxes
Source: Front Vet Sci. 2025 Apr 9;12:1567009. doi: 10.3389/fvets.2025.1567009 (PMC12016882; doi:10.3389/fvets.2025.1567009)
Supplement: Supplementary file 1 [file Table_1.docx]

|  |
| --- |

**Supplementary Table 1.** Primer sequences of Virulence genes.

| **Virulence gene** | **Forward (5′-3′)** | **Reverse (5′-3′)** | **Size (bp)** |
| --- | --- | --- | --- |
| *Vat* | TCCTGGGACATAATGGCTAG | GTGTCAGAACGGAATTGTC | 981 |
| *iutA* | GGCTGGACATCATGGGAACTGG | CGTCGGGAACGGGTAGAATCG | 302 |
| *Iss* | CAGCAACCCGAACCACTTGATG | AGCATTGCCAGAGCGGCAGAA | 323 |
| *hlyF* | GGCCACAGTCGTTTAGGGTGCTTACC | GGCGGTTTAGGCATTCCGATACTCAG | 450 |
| *iucD* | ACAAAAAGTTCTATCGCTTCC | CCTGATCCAGATGATGCTC | 714 |
| *tsh* | GTCTGTCAGACGTCTGTGTTTC | ATAGGATGACAGGCTACCGAC | 598 |
| *cvaA/B* | TGGTAGAATGTGCCAGAGCAAG | GAGCTGTTTGTAGCGAAGCC | 1181 |
| *cvaC* | GAAGCCACTCGTTCAAATC | CTGAAGCACCACCAGAAAC | 437 |
| *trat* | GGTGTGGTGCGATGAGCACAG | CACGGTTCAGCCATCCCTGAG | 290 |
| *ECs3737* | TACTAATGCCATATAGCCCCATAA | CTACGCTTTTAACAAACGATTGAT | 445 |
| *ECs370* | ATTGCCAAATAATGCCAGAAGAGTCACC | TCAATGTTGGACCGAATGTGAACGAATA | 581 |
