# Supplementary material for: Drug resistance and pathogenicity characteristics of Escherichia coli causing pneumonia in farmed foxes
Source: Front Vet Sci. 2025 Apr 9;12:1567009. doi: 10.3389/fvets.2025.1567009 (PMC12016882; doi:10.3389/fvets.2025.1567009)
Supplement: Supplementary file 2 [file Table_2.docx]

**Supplementary Table 2.** Primer sequences of antibiotic resistance genes.

| **Antibiotic classes** | **Gene** | **Primer sequence (5'→3')** | **Size (bp)** |
| --- | --- | --- | --- |
| *β*-Lactams | *CMY-2* | F:ACAGCCTCTTTCTCCACA  R:ATTGCCTCTTCGTAACTCA | 545 |
|  | *CTX-M* | F:GCTGGGTAAAGCATTGGG  R:TAGGTTGAGGCTGGGTGA | 224 |
|  | *SHV* | F:TGTATTATCTCCCTGTTAGC | 843 |
|  |  | R:TTAGCGTTGCCAGTGCTC |  |
| Quinolones | *gyrA* | F:CTGCGCGGCTGTGTTATAATT  R:CCGTGCCGTCATAGTTATCAA | 521 |
|  | *QnrS* | F:ACATAAAGACTTAAGTGATC  R:CAATTAGTCAGGATAAAC | 619 |
|  | *qnrD* | F:ACGGGGAATAGAGTTA  R:AATCAGCCAAAGACCAAT | 468 |
|  | *qnrC* | F:GGGTTGTACATTTATTGAATC  R:TCCACTTTACGAGGTTCT | 447 |
|  | *qepA* | F:CCAGCTCGGCAACTTGATAC  R:ATG CTCGCCTTCCAGAAAA | 570 |
|  | *qnrA* | F:CAGCAAGAGGATTTCTCA  R:GGCAGCACTATTACTCCCAA | 500 |
|  | *qnrB* | F:CCTGAGCGGCACTGAATTTAT  R:GTTTGCTGCTCGCCAGTCGA | 617 |
|  | *gyrB* | F:CTGCCGGGAAACTGGCAGA  R:TCGACGTCCGCATCGGTCAT | 299 |
| Macrolides | *mph(A)* | F:ATGACCGTAGTCACGACCG  R:TCATTCCGCTGCGGCGAGC | 906 |
| Tetracyclines | *tetB* | F:ATGAATAGTTCGACAAAGATCG | 1206 |
|  |  | R:CTAAGCACTTGTCTCCTGTTTAC |  |
|  | *tetA* | F:CACTATGGCATTCTGCTGGC | 344 |
|  |  | R:CATAGATCGCCGTGAAGAGG |  |
|  | *tetE* | F:TATTAACGGGCTGGCATTT  R:AGCTGTCAGGTGGGTCAAAC | 544 |
| Aminoglycosides | *strA* | F:GCGGATCCTTGAATCGAACTAATA | 803 |
|  |  | R:ACGTCGACTCAACCCCAAGTCAGAGG |  |
|  | *aadA2* | F:CATCCCGTGGCGTTATCC  R:CTGGGCAGGTAGGCGTTT | 370 |
|  | *aac2* | F:GGCAATAACGGAGGCAATTCGA | 698 |
|  |  | R:CTCGATGGCGACCGAGCTTCA |  |
|  | *aac4* | F:CGATGCTCTATGAGTGGCTAA  R:GTCCGTTTGGATCTTGGTGA | 356 |
| Sulfonamides | *sul* | F:TGGTGACGGTGTTCG GCATTC | 750 |
|  |  | R:GCGAAGGTTTCCGAGAAGGTG |  |
|  | *sul2* | F:ATGAATAAATCGCTCATCAT  R:TTAACGAATTCTTGCGGTTTC | 816 |
| Amphenicols | *floR* | F:ATGACCACCACACGCCCCG  R:CTAGACGACTGGCGACTTCT | 1215 |
| fosfomycin | *fosA* | F:ATGCTGCAGGGATTGAATCATC  R:TCAATCAAAAAAGACCATCC | 417 |
| integrons | *int1* | F:GGGTCAAGGATCTGGATTTCG | 475 |
|  |  | R:ACATGCGTGTAAATCATCGTCG |  |
|  | *Int2* | F:CACGGATATGCGACAAAAAGGT  R:GTAGCAAACGAGTGACGAAATG | 750 |

|  |
| --- |
