# Supplementary material for: Drug resistance and pathogenicity characteristics of Escherichia coli causing pneumonia in farmed foxes
Source: Front Vet Sci. 2025 Apr 9;12:1567009. doi: 10.3389/fvets.2025.1567009 (PMC12016882; doi:10.3389/fvets.2025.1567009)
Supplement: Supplementary file 3 [file Table_3.docx]

|  |
| --- |

| **Gene** | **Primer sequence (5'→3')** | **Size (bp)** | **Annealing temperature (°C)** |
| --- | --- | --- | --- |
| *adk* | F: ATTCTGCTTGGCGCTCCGGG  R: CCGTCAACTTTCGCGTATTT | 583 | 52 |
| *icd* | F:ATGGAAAGTAAAGTAGTTGTTCCGGCACA  R: GGACGCAGCAGGATCTGTT | 878 | 52 |
| *gyrB* | F: TCGGCGACACGGATGACGGC  R: ATCAGGCCTTCACGCGCATC | 911 | 58 |
| *recA* | F: CGCATTCGCTTTACCCTGACC  R: TCGTCGAAATCTACGGACCGGA | 780 | 58 |
| *mdh* | F:ATGAAAGTCGCAGTCCTCGGCGCTGCTGGCGG  R:TTAACGAACTCCTGCCCCAGAGCGATATCTTTCTT | 932 | 58 |
| *fumC* | F: TCACAGGTCGCCAGCGCTTC  R: GTACGCAGCGAAAAAAGATTC | 806 | 52 |
| *purA* | F: CGCGCTGATGAAAGAGATGA  R: CATACGGTAAGCCACGCAGA | 816 | 54 |

**Supplementary Table 3.** Primer sequences of housekeeping genes.
