# Supplementary material for: Drug resistance and pathogenicity characteristics of Escherichia coli causing pneumonia in farmed foxes
Source: Front Vet Sci. 2025 Apr 9;12:1567009. doi: 10.3389/fvets.2025.1567009 (PMC12016882; doi:10.3389/fvets.2025.1567009)
Supplement: Supplementary file 4 [file Table_4.docx]

**Supplementary Table 4.** Information on the antibiotic resistance of each *Escherichia coli* strain.

| **Strain name** | **Antibiotic resistance of strains** | **Quantity** | **Antibiotic classes** |
| --- | --- | --- | --- |
| EC-Cl-1 | AMP+TET+DOX+MINO  +CIP+IPM+LEV+AUG  +TMP+SUL+SMX | 11 | 4 |
| EC-Cl-2 | AMP+CFP+CTX+GEN  +TET+DOX+MINO+AZM  +NOR+OFX+CIP+IPM  +AUG+TMP+STREP  +SUL+SMX+TOB | 18 | 6 |
| EC-Cl-3 | AMP+TET+DOX+MINO  +NOR+CIP+IPM+LEV  +AUG+STREP+SUL | 11 | 5 |
| EC-Cl-4 | AMP+TET+DOX+MINO  +CIP+LEV+AUG  +STREP+SUL | 9 | 5 |
| EC-Cl-5 | AMP+CFP+CTX+TET  +CIP+IPM+AUG+TMP  +SUL+SMX | 10 | 4 |
| EC-Cl-6 | AMP+GEN+KAN+TET  +DOX+MINO+AUG+TMP  +SUL+SMX+TOB | 11 | 4 |
| EC-Cl-7 | AMP+GEN+KAN+TET  +DOX+MINO+NOR+OFX  +CIP+IPM+LEV+AUG+TMP  +STREP+SUL+SMX | 16 | 5 |
| EC-Cl-8 | AMP+CTX+TET+CIP  +IPM+AUG+TMP+SUL  +SMX | 9 | 4 |
| EC-Cl-9 | KAN+TET+DOX+MINO+IPM | 5 | 3 |
| EC-Cl-10 | AMP+AMK+GEN+KAN  +TET+DOX+AZM+NOR  +OFX+CIP+IPM+LEV  +AUG+TMP+STREP  +SUL+SMX+TOB+NET | 19 | 6 |
| EC-Cl-11 | KAN+TET+DOX | 3 | 2 |
| EC-Cl-12 | AMP+GEN+KAN+TET  +DOX+MINO+NOR+OFX  +CIP+IPM+LEV+TMP  +STREP+SUL+SMX | 15 | 5 |
| EC-Cl-13 | AMP+CAZ+CFP+CTX  +KAN+TET+DOX+MINO  +NOR+OFX+CIP+IPM  +LEV+FEP+AUG+TMP  +STREP+SUL+SMX | 19 | 5 |
| EC-Cl-14 | AMP+CAZ+CFP+CTX  +GEN+KAN+TET+CIP  +IPM+AUG+TMP+SUL  +SMX+TOB | 14 | 5 |
| EC-Cl-15 | AMP+GEN+KAN+TET  +DOX+MINO+NOR+OFX  +CIP+IPM+LEV+AUG  +TMP+STREP+SUL+SMX  +TOB | 17 | 5 |
| EC-Cl-16 | AMP+GEN+KAN+TET  +DOX+MINO+NOR+OFX  +CIP+IPM+LEV+AUG  +TMP+STREP+SUL+SMX | 16 | 5 |
| EC-Cl-17 | AMP+TET+DOX+MINO  +IPM+AUG+TMP+SMX | 8 | 3 |
| EC-Cl-18 | AMP+GEN+KAN+TET  +DOX+MINO+NOR+OFX  +CIP+LEV+AUG+TMP  +STREP+SUL+SMX+TOB | 16 | 5 |
| EC-Cl-19 | AMP+TET+DOX+MINO  +NOR+OFX+CIP+LEV  +TMP+STREP+SUL+SMX | 12 | 5 |
| EC-Cl-20 | KAN+TET+IPM | 3 | 3 |
| EC-Cl-21 | KAN+TET+DOX+IPM | 4 | 3 |
| EC-Cl-22 | AMP+CAZ+CFP+CTX  +KAN+TET+DOX+FEP  +AUG+TMP+SUL+SMX | 12 | 4 |
| EC-Cl-23 | AMP+TET+IPM+TMP  +SUL+SMX | 6 | 3 |
| EC-Cl-24 | AMP+GEN+KAN+TET  +DOX+TMP+SUL+SMX | 8 | 4 |
| EC-Cl-25 | KAN+TET+DOX | 3 | 2 |
| EC-Cl-26 | AMP+GEN+KAN+TET  +DOX+MINO+NOR+OFX  +CIP+LEV+TMP+STREP  +SUL+SMX | 14 | 5 |
| EC-Cl-27 | AMP+GEN+KAN+TET  +DOX+MINO+NOR+OFX  +CIP+LEV+TMP+STREP  +SUL+SMX+TOB | 15 | 5 |
| EC-Cl-28 | AMP+CAZ+CFP+CTX  +AMK+GEN+KAN+TET  +DOX+MINO+NOR+OFX  +CIP+LEV+FEP+AUG  +TMP+SMX+TOB+NET | 20 | 5 |
| EC-Cl-29 | AMP+CAZ+CFP+CTX  +AMK+GEN+KAN+TET  +DOX+MINO+NOR+OFX  +CIP+LEV+FEP+AUG  +TMP+STREP+SUL+SMX  +TOB+NET | 22 | 5 |
| EC-Cl-30 | AMP+KAN+TET+NOR  +OFX+CIP+IPM+LEV  +TMP+SUL+SMX | 11 | 5 |
| EC-Cl-31 | AMP+GEN+KAN+TET  +DOX+NOR+OFX+CIP  +IPM+LEV+AUG+TMP  +STREP+SUL+SMX+TOB | 16 | 5 |
| EC-Cl-32 | AMP+CAZ+CFP+CTX  +GEN+KAN+TET+DOX  +NOR+OFX+CIP+IPM  +LEV+FEP+AUG+TMP  +STREP+SUL+SMX | 19 | 5 |
| EC-FN-1 | AMP+CFP+CTX+GEN  +KAN+TET+NOR+CIP  +IPM+LEV+FEP+AUG  +TMP+STREP+SUL+SMX  +TOB | 17 | 5 |
| EC-FN-2 | KAN+TET | 2 | 2 |
| EC-FN-3 | AMP+TET+DOX+MINO  +CIP+IPM+AUG+STREP  +SUL | 9 | 5 |
| EC-FN-4 | KAN+TET+TMP+SUL  +SMX | 5 | 3 |
| EC-FN-5 | AMP+CAZ+CFP+CTX  +GEN+KAN+TET+DOX  +CIP+IPM+LEV+AUG  +TMP+SUL+SMX+TOB | 16 | 5 |
| EC-FN-6 | AMP+CFP+CTX+TET  +IPM+AUG+SUL+TOB | 8 | 4 |
| EC-FN-7 | AMP+CFP+CTX+GEN  +KAN+TET+DOX+NOR  +OFX+CIP+LEV+AUG  +TMP+SUL+SMX+TOB | 16 | 5 |
| EC-FN-8 | AMP+GEN+TET+DOX  +MINO+NOR+OFX+CIP  +IPM+LEV+AUG+TMP  +STREP+SUL+SMX | 15 | 5 |
| EC-FN-9 | AMP+GEN+TET+AUG  +TMP+SUL+SMX+TOB | 8 | 4 |
| EC-FN-10 | AMP+CFP+CTX+TET  +CIP+TMP+SUL+SMX | 8 | 4 |
| EC-FN-11 | AMP+GEN+TET+IPM  +AUG+TMP+SUL+SMX  +TOB | 9 | 4 |
| EC-FN-12 | AMP+CFP+CTX+TET  +CIP+AUG+TMP+SUL  +SMX | 9 | 4 |
| EC-FN-13 | AMP+CTX+GEN+KAN  +TET+NOR+OFX+CIP  +LEV+TMP+STREP+SUL  +SMX+TOB | 14 | 5 |
| EC-FN-14 | AMP+TET+DOX+MINO  +CIP+IPM+LEV+AUG  +STREP+SUL | 10 | 5 |
| EC-FN-15 | AMP+TET+DOX+MINO  +IPM+AUG | 6 | 2 |
| EC-FN-16 | AMP+CAZ+CFP+CTX  +KAN+TET+DOX+MINO  +NOR+OFX+CIP+LEV  +FEP+AUG+TMP+SUL  +SMX | 17 | 5 |
| EC-FN-17 | AMP+CFP+CTX+GEN  +TET+DOX+AZM+NOR  +OFX+CIP+LEV+AUG  +TMP+STREP+SUL+SMX  +TOB | 17 | 6 |
| EC-FN-18 | AMP+GEN+TET+IPM  +MEM+AUG+TMP+SUL  +SMX | 9 | 4 |
| EC-FN-19 | AMP+TET+DOX+MINO  +AUG+TMP+SUL+SMX | 8 | 3 |
| EC-FN-20 | AMP+KAN+TET+DOX  +MINO+NOR+OFX+CIP  +LEV+AUG+TMP+STREP  +SUL+SMX | 14 | 5 |
| EC-FN-21 | AMP+TET+DOX+MINO  +IPM+LEV+AUG+TMP  +STREP+SUL+SMX | 11 | 5 |
| EC-FN-22 | AMP+CFP+CTX+TET  +CIP+AUG+TMP+SUL  +SMX | 9 | 4 |
| EC-LL-1 | AMP+GEN+KAN+TET  +CIP+LEV+AUG+STREP  +SUL+ SMX | 10 | 5 |
| EC-LL-2 | AMP+CFP+CTX+GEN  +KAN+TET+DOX+MINO  +NOR+OFX+CIP+MEM  +LEV+AUG+TMP+STREP  +SUL+SMX | 18 | 5 |
| EC-LL-3 | AMP+CFP+CTX+TET  +DOX+MINO+NOR+OFX  +CIP+LEV+AUG+TMP  +SUL+SMX | 14 | 4 |
| EC-LL-4 | AMP+CFP+CTX+GEN  +KAN+TET+NOR+OFX  +CIP+LEV+AUG+TMP  +STREP+SUL+SMX+TOB | 16 | 5 |
| EC-LL-5 | AMP+KAN+TET+NOR  +OFX+CIP+LEV+AUG  +TMP+SUL+SMX | 11 | 5 |
| EC-LL-6 | AMP+CFP+CTX+GEN  +KAN+TET+DOX+NOR  +OFX+CIP+LEV+AUG  +TMP+STREP+SUL+SMX  +TOB | 17 | 5 |
| EC-LL-7 | AMP+CFP+CTX+KAN  +TET+AZM+NOR+OFX  +CIP+MEM+LEV+AUG  +TMP+STREP+SUL+SMX | 16 | 6 |
| EC-LL-8 | AMP+KAN+TET+NOR  +OFX+CIP+LEV+AUG  +TMP+SUL+SMX | 11 | 5 |
| EC-LL-9 | AMP+CAZ+CFP+CTX  +GEN+KAN+TET+DOX  +NOR+OFX+CIP+LEV  +AUG+TMP+STREP+SUL  +SMX | 17 | 5 |
| EC-LL-10 | AMP+CTX+GEN+KAN  +TET+DOX+NOR+OFX  +CIP+LEV+AUG+TMP  +STREP+SUL+SMX+TOB | 16 | 5 |
| EC-LT-1 | AMP+GEN+TET+DOX  +NOR+OFX+CIP+LEV  +AUG+TMP+STREP+SUL  +SMX | 13 | 5 |
| EC-LT-2 | AMP+CFP+CTX+KAN  +TET+AZM+NOR+OFX  +CIP+LEV+AUG+TMP  +STREP+SUL+SMX | 15 | 6 |
| EC-LT-3 | AMP+CFP+CTX+GEN  +KAN+TET+NOR+OFX  +CIP+LEV+AUG+TMP  +STREP+SUL+SMX+TOB | 16 | 5 |
| EC-LT-4 | AMP+CAZ+CFP+CTX  +GEN+KAN+TET+DOX  +NOR+OFX+CIP+LEV  +AUG+TMP+STREP+SUL  +SMX | 17 | 5 |
| EC-LT-5 | AMP+CFP+CTX+CIP  +LEV+AUG+TMP+STREP  +SUL+SMX | 10 | 4 |
| EC-LT-6 | AMP+CAZ+CFP+CTX  +TET+NOR+OFX+CIP  +LEV+AUG+TMP+STREP  +SUL+SMX | 14 | 5 |
| EC-LT-7 | AMP+CFP+CTX+GEN  +KAN+TET+NOR+OFX  +CIP+LEV+AUG+TMP  +STREP+SUL+SMX+TOB | 16 | 5 |
| EC-LT-8 | AMP+CFP+CTX+GEN  +KAN+TET+NOR+OFX  +CIP+LEV+AUG+TMP  +STREP+SUL+SMX+TOB | 16 | 5 |
| EC-LT-9 | AMP+CTX+GEN+KAN  +TET+DOX+NOR+OFX  +CIP+LEV+AUG+TMP  +STREP+SUL+SMX+TOB | 16 | 5 |
| EC-LT-10 | AMP+KAN+TET+DOX  +MINO+NOR+OFX+CIP  +LEV+AUG+TMP+STREP  +SUL+SMX | 14 | 5 |
| EC-LT-11 | AMP+CFP+CTX+CIP  +AUG+SUL | 6 | 3 |
| EC-LT-12 | TET+DOX | 2 | 1 |
| EC-LT-13 | TET+TMP+SUL+SMX | 4 | 2 |
| EC-LT-14 | TET+DOX | 2 | 1 |
| EC-QA-1 | TET+TMP+SUL+SMX | 4 | 2 |
| EC-QA-2 | TET+DOX+TMP+SUL  +SMX | 5 | 2 |
| EC-QA-3 | AMP+CAZ+CFP+CTX  +GEN+KAN+TET+DOX  +NOR+CIP+LEV+FEP  +AUG+TMP+STREP+SUL  +SMX | 17 | 5 |
| EC-QA-4 | TET+DOX | 2 | 1 |
| EC-QA-5 | TET+STREP+SUL | 3 | 3 |
| EC-LN-1 | TET+TMP+SUL+SMX | 4 | 2 |
| EC-LN-2 | AMP+CAZ+CFP+CTX  +TET+AZM+CIP+LEV  +AUG+TMP+STREP+SUL  +SMX | 13 | 6 |
| EC-LN-3 | TET+DOX | 2 | 1 |
| EC-LN-4 | TET+DOX | 2 | 1 |
| EC-LN-5 | TET+DOX | 2 | 1 |
| EC-LN-6 | TET+TMP+SUL+SMX | 4 | 2 |
| EC-FN-1 | AMP+CAZ+CFP+CTX  +GEN+KAN+TET+DOX  +NOR+OFX+CIP+LEV  +FEP+AUG+TMP+STREP  +SUL+SMX+TOB | 19 | 5 |
| EC-FN-2 | TET+DOX | 2 | 1 |
| EC-FN-3 | AMP+CFP+CTX+GEN  +TET+DOX+TMP+SUL  +SMX+TOB | 10 | 4 |
| EC-FN-4 | TET+DOX | 2 | 1 |
| EC-FN-5 | KAN+TET+DOX | 3 | 2 |
| EC-CFD-1 | TET+DOX | 2 | 1 |
| EC-CFD-2 | TET+DOX+STREP | 3 | 2 |
| EC-CFD-3 | TET+TMP+SUL+SMX | 4 | 2 |
| EC-CFD-4 | AMP+TET+TMP+SUL  +SMX | 5 | 3 |
| EC-CFD-5 | TET+DOX | 2 | 1 |
| EC-CFD-6 | TET+STREP+SUL | 3 | 3 |
| EC-CFD-7 | TET+DOX | 2 | 1 |

Note: ampicillin (AMP); Ceftazidime (CAZ); Cefoperazone (CFP); Cefotaxime (CTX); Amikacin (AMK); Gentamicin (GEN); Kanamycin (KAN); Tetracycline (TET); Doxycycline (DOX); Minocycline (MINO); Azithromycin (AZM); Norfloxacin (NOR); Ofloxacin (OFX); Ciprofloxacin (CIP); Imipenem (IPM); Meropenem (MEM); Levofloxacin (LEV); Cefepime (FEP); Augmentin(AUG); Trimethoprim(TMP); Streptomycin (STREP); Sulfisoxazole (SUL); Sulfamethoxazole(SMX); Tobramycin (TOB); Netilmicin (NET).
