# Supplementary material for: Drug resistance and pathogenicity characteristics of Escherichia coli causing pneumonia in farmed foxes
Source: Front Vet Sci. 2025 Apr 9;12:1567009. doi: 10.3389/fvets.2025.1567009 (PMC12016882; doi:10.3389/fvets.2025.1567009)
Supplement: Supplementary file 5 [file Table_5.docx]

**Supplementary Table 5.** Details of *Escherichia coli* strains that underwent MLST.

| **Strain Number** | **ST Type** | **Antibiotic resistance of strains** |
| --- | --- | --- |
| EC-Cl-3 | ST-101 | AMX+TET+DOX+MINO  +NOR+CIP+IPM+LEV  +AUG+STREP+SUL |
| EC-Cl-11 | ST-7203 | KAN+TET+DOX |
| EC-Cl-14 | ST-38 | AMX+CAZ+CFP+CTX  +GEN+KAN+TET+CIP  +IPM+AUG+TMP+SUL  +SMX+TOB |
| EC-Cl-15 | ST-744 | AMX+GEN+KAN+TET  +DOX+MINO+NOR+OFX  +CIP+IPM+LEV+AUG  +TMP+STREP+SUL+SMX  +TOB |
| EC-Cl-20 | ST-7203 | KAN+TET+IPM |
| EC-Cl-28 | ST-48 | AMX+CAZ+CFP+CTX  +AMK+GEN+KAN+TET  +DOX+MINO+NOR+OFX  +CIP+LEV+FEP+AUG  +TMP+SMX+TOB+NET |
| EC-FN-5 | ST-38 | AMX+CAZ+CFP+CTX  +GEN+KAN+TET+DOX  +CIP+IPM+LEV+AUG  +TMP+SUL+SMX+TOB |
| EC-FN-13 | ST-224 | AMX+CTX+GEN+KAN  +TET+NOR+OFX+CIP  +LEV+TMP+STREP+SUL  +SMX+TOB |
| EC-FN-20 | ST-410 | AMX+KAN+TET+DOX  +MINO+NOR+OFX+CIP  +LEV+AUG+TMP+STREP  +SUL+SMX |
| EC-LL-3 | ST-101 | AMX+CFP+CTX+TET  +DOX+MINO+NOR+OFX  +CIP+LEV+AUG+TMP  +SUL+SMX |
| EC-LT-4 | ST-410 | AMX+CAZ+CFP+CTX  +GEN+KAN+TET+DOX  +NOR+OFX+CIP+LEV  +AUG+TMP+STREP+SUL  +SMX |
| EC-LT-6 | ST-3285 | AMX+CAZ+CFP+CTX  +TET+NOR+OFX+CIP  +LEV+AUG+TMP+STREP  +SUL+SMX |
| EC-LT-9 | ST-224 | AMX+CTX+GEN+KAN  +TET+DOX+NOR+OFX  +CIP+LEV+AUG+TMP  +STREP+SUL+SMX+TOB |
| EC-LT-11 | ST-7584 | AMX+CFP+CTX+CIP  +AUG+SUL |
| EC-QA-2 | ST-127 | TET+DOX+TMP+SUL  +SMX |
| EC-QA-3 | ST-101 | AMX+CAZ+CFP+CTX  +GEN+KAN+TET+DOX  +NOR+CIP+LEV+FEP  +AUG+TMP+STREP+SUL  +SMX |
| EC-LN-2 | ST-127 | AMX+CAZ+CFP+CTX  +TET+AZM+CIP+LEV  +AUG+TMP+STREP+SUL  +SMX |
| EC-FN-1 | ST-101 | AMX+CAZ+CFP+CTX  +GEN+KAN+TET+DOX  +NOR+OFX+CIP+LEV  +FEP+AUG+TMP+STREP  +SUL+SMX+TOB |
| EC-CFD-4 | ST-23 | AMX+TET+TMP+SUL  +SMX |
